# True- and pseudo-mitral annular disjunction in patients undergoing cardiovascular magnetic resonance

**DOI:** 10.1016/j.jocmr.2024.101413

**Published:** 2024-12-30

**Authors:** Kamil Stankowski, Federica Catapano, Dario Donia, Renato Maria Bragato, Pedro Lopes, João Abecasis, António Ferreira, Leandro Slipczuk, Pier-Giorgio Masci, Gianluigi Condorelli, Marco Francone, Stefano Figliozzi

**Affiliations:** a IRCCS Humanitas Research Hospital, Milano, Italy; b Department of Biomedical Sciences, Humanitas University, Milano, Italy; c Cardiology Department, Hospital de Santa Cruz, Lisboa, Portugal; d Montefiore Health System, Cardiology Division, Bronx, New York, USA; e School of Biomedical Engineering and Imaging Sciences, King's College London, London, United Kingdom

**Keywords:** Mitral valve prolapse, Pseudo-MAD, True-MAD, Spatial resolution, CMR, Multimodality imaging

## Abstract

**Background:**

Mitral annular disjunction (MAD) is a controversial entity. Recently, a distinction between pseudo-MAD, present in systole and secondary to juxtaposition of the billowing posterior leaflet on the left atrial wall, and true-MAD, where the insertion of the posterior leaflet is displaced on the atrial wall both in diastole or in systole, has been proposed. We investigated the prevalence of pseudo-MAD and true-MAD.

**Methods:**

This was a retrospective study, including consecutive patients referred to cardiovascular magnetic resonance (CMR). MAD was defined as a ≥1 mm displacement between the left atrial wall-mitral valve leaflet junction hinge and the top of the left ventricular wall, measured from cine-CMR images in the three long-axis views. Pseudo-MAD and true-MAD were defined as the presence of MAD only in systole or both in systole and diastole, respectively.

**Results:**

Two hundred and ninety patients (59 [47–71] years; 181/290 men, 62%) were included. Mitral valve prolapse (MVP) and MAD were found in 24/290 (8%) and 145/290 (50%) patients, of which 100/290 (35%) with true-MAD and 45/290 (16%) with pseudo-MAD. In all measurements, systolic MAD extent (2.3 [1.7–3.0] mm) resulted equal to or greater than diastolic MAD extent (2.0 [1.5–2.9] mm). The most frequent MAD location was the inferior wall (117/290, 40%) and the inferolateral wall was the rarest (50/290, 17%). In patients with MVP, the prevalence of MAD was higher (21/24, 88%), mainly driven by a higher prevalence of pseudo-MAD, as the prevalence of true-MAD did not vary significantly in patients with vs without MVP (*p* = 0.22), except for the inferolateral wall (9/24, 38% vs 20/266, 8%; *p* < 0.001). The extent of pseudo-MAD was greater in patients with MVP (4.0 [3.0–5.6] mm) than in those without MVP (2.0 [1.5–3.0]; *p* < 0.001), whereas the extent of true-MAD did not differ significantly (2.5 [2.0–3.2] mm and 1.9 [1.5–2.9] mm; *p* = 0.06). At the inferolateral wall, the prevalence of pseudo-MAD was 7/24, 29% vs 14/266, 5% (*p* < 0.001) in patients with vs without MVP.

**Conclusion:**

True-MAD was a common imaging finding in patients undergoing CMR, irrespective of MVP. Patients with MVP showed higher prevalence and extent of pseudo-MAD in all locations and true-MAD in the inferolateral wall

## Graphical abstract

**Figure:**
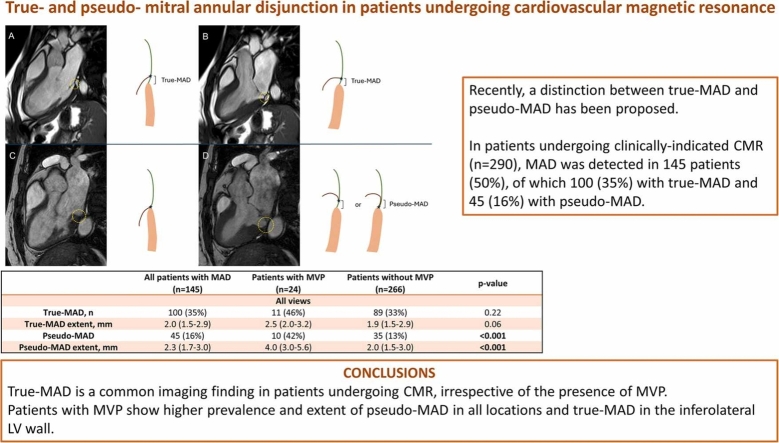

Mitral annular disjunction (MAD) is a controversial imaging entity with uncertain clinical significance, which has been either considered a pathological entity potentially related to mitral valve prolapse (MVP) and sudden cardiac death [1], [2] or, on the opposite, a normal, benign finding [3], [4], [5]. Cardiovascular magnetic resonance (CMR) is poised to be the ideal imaging modality to assess MAD [3], and research endeavors are needed to distinguish the benign characteristics of MAD from potentially malignant features. Recently, a distinction between pseudo-MAD, present only in systole and secondary to a juxtaposition of the belly of the billowing posterior leaflet on the adjacent left atrial wall, and true-MAD, where the insertion of the posterior leaflet is clearly displaced on the atrial wall both in diastole or systole, has been proposed [6]. Also, in a subgroup of patients with pseudo-MAD, a limited MAD might be hidden during diastole because of low pressures in the left ventricle, only becoming evident during systolic contraction. We aimed to investigate the prevalence of pseudo-MAD and true-MAD in patients undergoing clinically indicated CMR.

This was a single-center retrospective study of prospectively collected data, including consecutive patients referred to CMR at IRCCS Humanitas Research Hospital, Milan, Italy from September to November 2023. The study inclusion criteria were i) absence of contraindication to CMR and ii) feasibility of MAD assessment. Patients with prior mitral valve surgery were excluded. The institutional review board approved this study. CMR scans were acquired using a 1.5T scanner (MAGNETOM Aera; Siemens Healthcare, Erlangen, Germany). A standardized acquisition protocol was carried out as per clinical needs, including cine sequences in two-chamber, three-chamber, and four-chamber orientations. Images were analyzed through a Circle CVI42 station-version 5.13.7 (Circle Cardiovascular Imaging Inc., Calgary, Alberta, Canada) according to current recommendations [7]. An experienced operator (S.F., EACVI CMR level 3) blinded to clinical data searched for MVP and MAD in standard long-axis views. MVP was defined as a systolic displacement ≥2 mm of one or both mitral valve leaflets above the annulus in three-chamber view [3], [4], [8]. True-MAD was defined as a separation ≥1 mm between the left atrial wall-mitral valve leaflet junction and the basal left ventricular (LV) wall during end-diastole and end-systole. Pseudo-MAD was defined as a separation ≥1 mm between the left atrial wall-mitral valve leaflet junction and the basal LV wall during end-systole only [3], [5], [6]. Both true- and pseudo-MAD extents were defined as the maximum longitudinal displacement in any long-axis view in end-diastole and end-systole, respectively [3], [4], [6]. In three-chamber and four-chamber views, the basal LV septal wall was excluded from MAD analysis due to the presence of the mitro-aortic curtain and the absence of left atrial wall above the myocardium [1], [4]. For the assessment of intra- and inter-observer reproducibility of MAD measurements, 30 randomly chosen subjects were re-analyzed by the same operator and by a second, less experienced, operator (D.D.; 6 months’ experience in CMR), blinded to the results of the first. The Shapiro-Wilk test was used to check the variables’ distribution. Continuous variables were expressed as mean ± standard deviation or median (25th/75th percentiles), as appropriate. Categorical variables were reported as numbers and percentages. Continuous variables were compared by means of the independent Student’s t-test and Mann-Whitney test, as appropriate, while categorical data by means of chi-square test. Intra- and inter-observer reproducibilities were evaluated by using two-way mixed intraclass-correlation-coefficient (ICC), Bland-Altman analysis, Spearman correlation coefficient (ρ), and Cohen's kappa coefficient. Data analysis was performed using Stata, version 18 (Stata Corp, College Station, Texas ). All reported *p*-values were two-sided and statistical significance was set at *p* < 0.05.

Among 302 available CMR studies, 10 and 2 studies were, respectively, excluded because of unfeasible MAD analysis due to low image quality and previous mitral surgery. Two hundred and ninety patients (59 [47–71] years; 181/290 men, 62%) were included. The indications of CMR were non-ischemic cardiomyopathy in 161/290 (56%), ischemic heart disease in 103/290 (36%), and other in 26/290 (9%). MVP and MAD were, respectively, found in 24/290 (8%) and 145/290 (50%) patients, of which 100/290 (35%) with true-MAD and 45/290 (16%) with pseudo-MAD. In all measurements, systolic MAD extent (2.3 [1.7–3.0] mm) resulted equal to or greater than diastolic MAD extent (2.0 [1.5–2.9] mm). The most frequent MAD location was the LV inferior wall in the two-chamber view (117/290; 40%), whereas the rarest one was the inferolateral wall in the three-chamber view (50/290, 17%). In patients with MVP, the prevalence of MAD was higher when considering all long-axis views (21/24, 88%), of which 11/24 (46%) presented with true-MAD and 10/24 (42%) with pseudo-MAD. This finding was mainly driven by a higher prevalence of pseudo-MAD in patients with MVP, as the prevalence of true-MAD did not vary significantly in patients with vs without MVP (*p* = 0.22), except for the inferolateral wall (9/24, 38% vs 20/266, 8%, *p* < 0.001, respectively). The extent of pseudo-MAD was greater in patients with MVP (4.0 [3.0–5.6] mm) than in those without MVP (2.0 [1.5–3.0] mm; *p* < 0.001) in all locations. On the contrary, the extent of true-MAD did not differ significantly between subjects with MVP (2.5 [2.0–3.2] mm) and without MVP (1.9 [1.5–2.9] mm; *p* = 0.06) (Table 1). Accordingly, in a secondary analysis employing a 2-mm cut-off for MAD identification, MAD was found in 94/290 (32%) subjects, of which 50/290 (17%) with true-MAD and 44/290 (15%) with pseudo-MAD (Supplementary Table 1). At the level of the inferolateral wall, the prevalence of pseudo-MAD was 7/24, 29% vs 14/266, 5% (*p* < 0.001) in patients with vs without MVP (Table 1).

**Table 1:** True- and pseudo-MAD characteristics in patients with and without MVP.

	All patients with MAD (n = 145)	Patients with MVP (n = 24)	Patients without MVP (n = 266)	*p*-value*
**All views**				
True-MAD, n	100/290 (35%)	11/24 (46%)	89/266 (33%)	0.22
True-MAD extent, mm	2.0 (1.5–2.9)	2.5 (2.0–3.2)	1.9 (1.5–2.9)	0.06
Pseudo-MAD, n	45/290 (16%)	10/24 (42%)	35/266 (13%)	**<0.001**
Pseudo-MAD extent, mm	2.3 (1.7–3.0)	4.0 (3.0–5.6)	2.0 (1.5–3.0)	**<0.001**
**Three-chamber view**				
True-MAD, n	29/290 (10%)	9/24 (38%)	20/266 (8%)	**<0.001**
True-MAD extent, mm	1.9 (1.3–2.5)	2.2 (1.9–2.5)	1.5 (1.2–2.6)	0.14
Pseudo-MAD, n	21/290 (7%)	7/24 (29%)	14/266 (5%)	**<0.001**
Pseudo-MAD extent, mm	2.0 (1.5–3.0)	3.3 (2.2–4.9)	1.7 (1.3–2.2)	**<0.001**
**Two-chamber view, anterior wall**				
True-MAD, n	46/290 (16%)	3/24 (13%)	43/266 (16%)	0.64
True-MAD extent, mm	2.0 (1.5–2.5)	2.5 (1.6–3.2)	2.0 (1.5–2.5)	0.35
Pseudo-MAD, n	30/290 (10%)	8/24 (33%)	22/266 (8%)	**<0.001**
Pseudo-MAD extent, mm	2.0 (1.5–2.9)	2.6 (1.6–3.3)	2.0 (1.5–2.7)	0.38
**Two-chamber view, inferior wall**				
True-MAD, n	76/290 (26%)	7/24 (29%)	69/266 (26%)	0.73
True-MAD extent, mm	2.0 (1.5–2.8)	2.2 (1.5–3.2)	2.0 (1.5–2.5)	0.30
Pseudo-MAD, n	41/290 (14%)	11/24 (46%)	30/266 (11%)	**<0.001**
Pseudo-MAD extent, mm	2.0 (1.6–3.0)	3.1 (2.3–4.3)	2.0 (1.5–2.8)	**0.003**
**Four-chamber view**				
True-MAD, n	32/290 (11%)	4/24 (17%)	28/266 (11%)	0.36
True-MAD extent, mm	1.5 (1.2–2.0)	2.9 (1.9–4.5)	1.5 (1.2–2.0)	0.13
Pseudo-MAD, n	33/290 (11%)	14/24 (58%)	19/266 (7%)	**<0.001**
Pseudo-MAD extent, mm	2.0 (1.3–2.9)	3.0 (2.0–3.9)	1.7 (1.2–2.3)	**<0.001**

*MAD* mitral annular disjunction, *MVP* mitral valve prolapse, *n* number of patients. Data are numbers (%) of cases, or medians (interquartile range). Bold values are statistically significant.

* *p*-value refers to the comparison of patients with and without MVP

Intra-observer reproducibility was good for true-MAD detection (Cohen’s kappa = 0.85), excellent for pseudo-MAD detection (Cohen’s kappa = 0.93), and excellent for MAD extent (ICC = 0.90 for both true- and pseudo-MAD). Inter-observer reproducibility was moderate for MAD detection (Cohen’s kappa = 0.72 for both true- and pseudo-MAD) and MAD extent (ICC = 0.67 for both). The complete per-view assessment is detailed in Supplementary Tables 2 and 3.

The main findings of the present study are i) true-MAD is a common imaging finding in patients undergoing CMR, thus probably not resulting in a distinctive abnormal feature related to MVP or sudden cardiac death; ii) patients with MVP show higher prevalence and extent of pseudo-MAD in all locations and true-MAD in the inferolateral LV wall.

Methodological discrepancies affect MAD assessment. Most published studies have measured MAD in end-systole [1], [3], [4], [5]. However, evaluating the systolic timeframes only does not allow distinguishing a true- from a pseudo-MAD, which may indeed be two different entities with potentially diverse clinical implications. The high prevalence of a true-MAD of a limited extent in the whole population has practical implications by framing this imaging entity as a normal variant of the mitral annulus, reflecting the extension of fibers into the mitral annulus from the contiguous fibrous trigones [9]. In fact, true-MAD was most common near the fibrous trigones (i.e., inferior and anterior walls in two-chamber view) and its presence did not differ in patients with and without MVP. However, true-MAD was more prevalent in patients with MVP at the level of the inferolateral wall. MAD at this site, although of limited extent, has been considered abnormal and associated with advanced degeneration of the mitral apparatus in the context of MVP [3], [5]. Our reproducibility analysis suggests that inter-observer variability is not negligible for MAD detection and calls for education and standardization in this imaging analysis.

The greater prevalence of pseudo-MAD in patients with MVP suggests that the redundant prolapsing leaflets can often generate the false appearance of a disjunction due to the sliding of the posterior leaflet on the atrial wall, when the mitral leaflet insertion is still at the ventricular myocardium basal point [6] (Fig. 1). Although our findings refute the sole presence of a true-MAD as a risk marker of sudden cardiac death in line with the high prevalence in this unselected cohort of patients, the present study is limited by the lack of analysis of ventricular arrhythmias or clinical follow-up, and larger longitudinal investigations remain needed to identify potential arrhythmogenic characteristics of MAD.

**Fig. 1:**
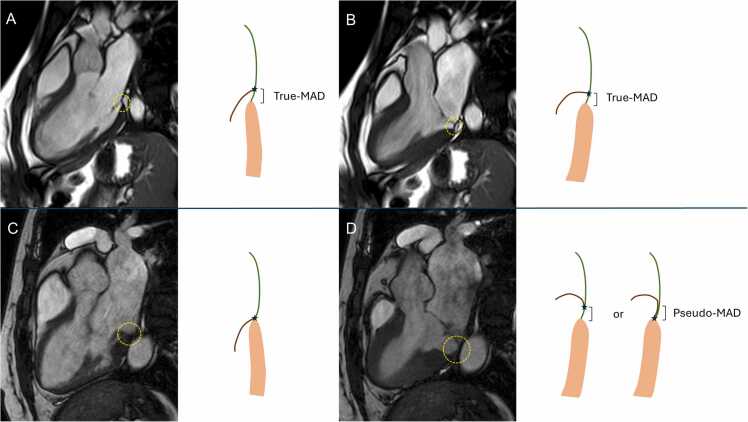
(A, B) End-diastolic (left) and end-systolic (right) cine-CMR frames and corresponding illustration showing MAD persisting throughout the cardiac cycle (true-MAD). (C, D) End-diastolic (left) and end-systolic (right) cine-CMR frames and corresponding illustration showing presence of disjunction only in systole (pseudo-MAD). It is not clear whether this results from sliding of the mitral annulus-ventricular junction in systole or from juxtaposition of the billowing posterior leaflet on the atrial wall. Green: atrial wall; brown: posterior mitral leaflet; pink: myocardial wall; star: mitral annulus insertion on the basal myocardial wall/atrial wall. *CMR* cardiovascular magnetic resonance, *MAD* mitral annular disjunction

## Funding

This work was partially supported by "Ricerca Corrente" funding from the Italian Ministry of Health to IRCCS Humanitas Research Hospital.

## Author contributions

João Abecasis: Writing—review and editing. Pedro Lopes: Writing—review and editing. Leandro Slipczuk: Writing—review and editing. António Ferreira: Writing—review and editing, Supervision. Gianluigi Condorelli: Writing—review and editing, Supervision. Pier-Giorgio Masci: Writing—review and editing, Supervision, Methodology, Conceptualization. Stefano Figliozzi: Writing—review and editing, Writing—original draft, Formal analysis, Data curation, Conceptualization. Kamil Stankowski: Writing—review and editing, Writing—original draft, Formal analysis, Data curation. Marco Francone: Writing—review and editing, Supervision. Federica Catapano: Writing—review and editing, Data curation. Renato Maria Bragato: Writing—review and editing, Supervision. Dario Donia: Writing—review and editing, Data curation.

## Ethics approval and consent

The authors confirm that patient consent is not applicable to this article as it is a retrospective study and the Institutional Review Board did not require consent from the patient.

## Declaration of competing interests

The authors declare that they have no known competing financial interests or personal relationships that could have appeared to influence the work reported in this paper.
