# Supplementary material for: True- and pseudo-mitral annular disjunction in patients undergoing cardiovascular magnetic resonance
Source: J Cardiovasc Magn Reson. 2024 Dec 30;27(1):101413. doi: 10.1016/j.jocmr.2024.101413 (PMC11786680; doi:10.1016/j.jocmr.2024.101413)
Supplement: Supplementary file 1 — Supplementary material [file mmc1.docx]

**SUPPLEMENTARY MATERIAL**

**Supplementary Table 1.** True and pseudo-MAD prevalence according to a 2 mm cut-off in patients with and without MVP.

**Supplementary Table 2.** Intra-observer reproducibility of MAD assessment.

**Supplementary Table 3.** Inter-observer reproducibility of MAD assessment.

**Supplementary Table 1. True and pseudo-MAD prevalence according to a 2 mm cut-off in patients with and without MVP.**

|  | **All patients with MAD ≥ 2 mm (n=94)** | **Patients with MVP**  **(n=24)** | **Patients without MVP**  **(n=266)** | **p-value*** |
| --- | --- | --- | --- | --- |
| ***All views*** | | | | |
| True-MAD, n | 50 (17%) | 9 (38%) | 41 (15%) | **0.006** |
| Pseudo-MAD, n | 44 (15%) | 12 (50%) | 32 (12%) | **<0.001** |
| ***Three-chamber view*** | | | | |
| True-MAD, n | 13 (4%) | 6 (25%) | 7 (3%) | **<0.001** |
| Pseudo-MAD, n | 13 (4%) | 7 (29%) | 6 (2%) | **<0.001** |
| ***Two-chamber view, anterior wall*** | | | | |
| True-MAD, n | 21 (7%) | 2 (8%) | 19 (7%) | 0.83 |
| Pseudo-MAD, n | 20 (7%) | 4 (17%) | 16 (6%) | **0.049** |
| ***Two-chamber view, inferior wall*** | | | | |
| True-MAD, n | 37 (13%) | 4 (17%) | 33 (12%) | 0.55 |
| Pseudo-MAD, n | 34 (12%) | 11 (46%) | 23 (9%) | **<0.001** |
| ***Four-chamber view*** | | | | |
| True-MAD, n | 11 (4%) | 3 (13%) | 8 (3%) | **0.02** |
| Pseudo-MAD, n | 26 (9%) | 13 (54%) | 13 (5%) | **<0.001** |

MAD: mitral annular disjunction; MVP: mitral valve prolapse; n: number of patients. *p-value refers to the comparison of patients with and without MVP.

**Supplementary Table 2. Intra-observer reproducibility of MAD assessment.**

|  | ***Spearman ρ*** | ***Cohen’s K Coefficient*** | ***Bias*** | ***Upper LOA*** | ***Lower LOA*** | ***ICC*** |
| --- | --- | --- | --- | --- | --- | --- |
| ***All views*** | | | | | | |
| ***True-MAD presence*** | 0.93 | 0.85 | - | - | - | - |
| ***True-MAD extent*** | 0.89 | - | 0.20 | 1.87 | -1.47 | 0.90 (0.81-0.95) |
| ***Pseudo-MAD presence*** | 0.86 | 0.93 | - | - | - | - |
| ***Pseudo-MAD extent*** | 0.88 | - | -0.15 | 1.86 | -2.17 | 0.90 (0.80-0.95) |
| ***Three-chamber view*** | | | | | | |
| ***True-MAD presence*** | 1 | 1 | - | - | - | - |
| ***True-MAD extent*** | 1 | - | 0.06 | 0.70 | -0.58 | 0.93 (0.86-0.96) |
| ***Pseudo-MAD presence*** | 0.52 | 0.52 | - | - | - | - |
| ***Pseudo-MAD extent*** | 0.51 | - | -0.04 | 2.61 | -2.70 | 0.55 (0.24-0.75) |
| ***Two-chamber view, anterior wall*** | | | | | | |
| ***True-MAD presence*** | 1 | 1 | - | - | - | - |
| ***True-MAD extent*** | 1 | - | 0 | 0.54 | -0.56 | 0.98 (0.96-0.99) |
| ***Pseudo-MAD presence*** | 0.68 | 0.66 | - | - | - | - |
| ***Pseudo-MAD extent*** | 0.76 | - | -0.46 | 2.30 | -3.23 | 0.76 (0.56-0.88) |
| ***Two-chamber view, inferior wall*** | | | | | | |
| ***True-MAD presence*** | 0.90 | 0.90 | - | - | - | - |
| ***True-MAD extent*** | 0.88 | - | 0.15 | 1.61 | -1.30 | 0.89 (0.79-0.94) |
| ***Pseudo-MAD presence*** | 0.92 | 0.87 | - | - | - | - |
| ***Pseudo-MAD extent*** | 0.91 | - | 0 | 1.85 | -1.84 | 0.91 (0.83-0.96) |
| ***Four-chamber view*** | | | | | | |
| ***True-MAD presence*** | 1 | 1 | - | - | - | - |
| ***True-MAD extent*** | 0.99 | - | 0 | 0.28 | -0.28 | 0.98 (0.96-0.99) |
| ***Pseudo-MAD presence*** | 0.70 | 0.70 | - | - | - | - |
| ***Pseudo-MAD extent*** | 0.72 | - | 0.17 | 2.21 | -1.87 | 0.71 (0.48-0.85) |

ICC: intraclass correlation coefficient; LOA: limits of agreement; MAD: mitral annular disjunction.

**Supplementary Table 3. Inter-observer reproducibility of MAD assessment.**

|  | ***Spearman ρ*** | ***Cohen’s K Coefficient*** | ***Bias*** | ***Upper LOA*** | ***Lower LOA*** | ***ICC*** |
| --- | --- | --- | --- | --- | --- | --- |
| ***All views*** | | | | | | |
| ***True-MAD presence*** | 0.73 | 0.72 | - | - | - | - |
| ***True-MAD extent*** | 0.64 | - | -0.6 | 0.64 | -0.78 | 0.67 (0.41-0.82) |
| ***Pseudo-MAD presence*** | 0.73 | 0.72 | - | - | - | - |
| ***Pseudo-MAD extent*** | 0.66 | - | -1.21 | 2.18 | -4.61 | 0.67 (0.42-0.83) |
| ***Three-chamber view*** | | | | | | |
| ***True-MAD presence*** | 0.69 | 0.65 | - | - | - | - |
| ***True-MAD extent*** | 0.71 | - | -0.06 | 1.25 | -1.37 | 0.67 (0.42-0.83) |
| ***Pseudo-MAD presence*** | 0.89 | 0.88 | - | - | - | - |
| ***Pseudo-MAD extent*** | 0.92 | - | -0.12 | 1.35 | -1.59 | 0.81 (0.64-0.90) |
| ***Two-chamber view, anterior wall*** | | | | | | |
| ***True-MAD presence*** | 0.67 | 0.66 | - | - | - | - |
| ***True-MAD extent*** | 0.63 | - | -0.16 | 2.15 | -2.48 | 0.67 (0.42-0.83) |
| ***Pseudo-MAD presence*** | 0.57 | 0.57 | - | - | - | - |
| ***Pseudo-MAD extent*** | 0.66 | - | -0.63 | 2.26 | -3.54 | 0.65 (0.38-0.81) |
| ***Two-chamber view, inferior wall*** | | | | | | |
| ***True-MAD presence*** | 0.67 | 0.66 | - | - | - | - |
| ***True-MAD extent*** | 0.72 | - | -0.17 | 2.02 | -2.37 | 0.73 (0.51-0.86) |
| ***Pseudo-MAD presence*** | 0.73 | 0.73 | - | - | - | - |
| ***Pseudo-MAD extent*** | 0.77 | - | -1.08 | 2.07 | -4.24 | 0.68 (0.43-0.83) |
| ***Four-chamber view*** | | | | | | |
| ***True-MAD presence*** | 0.85 | 0.68 | - | - | - | - |
| ***True-MAD extent*** | 0.87 | - | 0.04 | 0.50 | -0.41 | 0.96 (0.92-0.98) |
| ***Pseudo-MAD presence*** | 0.62 | 0.62 | - | - | - | - |
| ***Pseudo-MAD extent*** | 0.69 | - | -0.26 | 1.57 | -2.09 | 0.74 (0.52-0.86) |

ICC: intraclass correlation coefficient; LOA: limits of agreement; MAD: mitral annular disjunction.
